# A Diagnostic Panel for Acquired Immune-Mediated Polyneuropathies Based on the Expression of lncRNAs

**DOI:** 10.3389/fimmu.2021.643615

**Published:** 2021-02-23

**Authors:** Bashdar Mahmud Hussen, Fwad Nicknafs, Hazha Jamal Hidayat, Arezou Sayad, Soudeh Ghafouri-Fard, Mohammad Taheri

**Affiliations:** ^1^Pharmacognosy Department, College of Pharmacy, Hawler Medical University, Erbil, Iraq; ^2^Department of Medical Genetics, School of Medicine, Shahid Beheshti University of Medical Sciences, Tehran, Iran; ^3^Department of Biology, College of Education, Salahaddin University-Erbil, Erbil, Iraq; ^4^Urology and Nephrology Research Center, Shahid Beheshti University of Medical Sciences, Tehran, Iran

**Keywords:** AIDP, CIDP, lncRNA, ANRIL, MALAT1, CCAT1, CCAT2, CCHE1

## Abstract

Long non-coding RNAs (lncRNAs) have been shown to alter immune responses, thus contributing to the pathobiology of autoimmune conditions. We investigated the expression levels of ANRIL, PICART1, MALAT1, CCAT1, CCAT2, and CCHE1 lncRNAs in acute and chronic inflammatory demyelinating polyneuropathy (AIDP and CIDP). ANRIL, PICART1, MALAT1, CCAT1, CCAT2, and CCHE1 lncRNAs were significantly downregulated in individuals with both AIDP and CIDP compared with unaffected individuals. Gender-based comparisons also verified such downregulations in both male and female subjects compared with sex-matched unaffected controls for all lncRNAs. There was no significant difference in the expression of any of the lncRNAs between cases with AIDP and cases with CIDP. While the expression levels of ANRIL and PICART1 were significantly correlated in healthy subjects (r = 0.86, *p* = 8.5E-16), similar analysis in cases with AIDP and CIDP revealed no significant correlation. The most robust correlation among patients was detected between ANRIL and MALAT1 lncRNAs (r = 0.59, *p* = 3.52E-6). ANRIL, MALAT1, and PICART1 had the diagnostic power of 0.96, 0.94, and 0.92 in distinguishing between cases with CIDP and controls, respectively. A combination of all lncRNAs resulted in 0.95 diagnostic power with a sensitivity of 0.85 and specificity of 0.96 for this purpose. Diagnostic power values of these lncRNAs in differentiation between cases with AIDP and controls were 0.98, 0.95, and 0.93, respectively. The combinatorial diagnostic power reached 0.98 for differentiation between cases with AIDP and controls. The six-lncRNA panel could differentiate combined cases with AIDP and CIDP from controls with area under the curve (AUC), sensitivity, and specificity values of 0.97, 0.90, and 0.96, respectively. Collectively, the lncRNA panel is suggested as a sensitive and specific diagnostic panel for acquired immune-mediated polyneuropathies.

## Introduction

Immune-mediated polyneuropathies comprise a wide spectrum of disorders with variable subtypes including the acute monophasic form, i.e., acute inflammatory demyelinating polyneuropathy (AIDP) and the chronic, corticosteroid-responsive type, namely, chronic inflammatory demyelinating polyneuropathy (CIDP). These conditions are thought to result from autoimmune responses against some elements of the myelin sheath of peripheral nerves or some other proteins (1). Yet, there is no constant presence of autoantibodies in all subtypes (1). The existence of several subtypes has made the diagnosis challenging (1). Currently, the diagnosis of these conditions is based on the assessment of protein levels in the cerebrospinal fluid (CSF), nerve biopsy, and electrodiagnostic methods (2). Most diagnostic criteria for these conditions propose evidence to support the diagnosis, instead of conclusive confirmation of the diagnosis (2). Therefore, it is necessary to identify diagnostic biomarkers for routine application in the clinical setting. A large body of evidence supports the putative role of long non-coding RNAs (lncRNAs) as biomarkers for autoimmune disorders (3, 4). A number of lncRNAs are more promising in this field, as they regulate immune responses from different points. For instance, ANRIL expression has been shown to be regulated by nuclear factor kappa B (NF-κB) through tumor necrosis factor alpha (TNF-α). This lncRNA regulates the expression of a number of proinflammatory proteins (5). PICART1 has been shown to suppress JAK2/STAT3 signaling (6). STAT3 has functional roles in the regulation of immune response through its interplay with IRF4, BATF, and RORγt (7). MALAT1 regulates the maturation and apoptosis of dendritic cells (DCs) and the production of proinflammatory cytokine by these cells (8). CCAT1 can control the expression of interleukin-10 (IL-10) and modulate macrophage polarization (9). CCAT2 has functional interactions with TGF-β signaling (10), a pathway that has multiple roles in the regulation of immune responses through the regulation of thymic T-cell selection, the preservation of balance of the naïve T-cell population, the suppression of cytotoxic T and T helper cells differentiation, and the enhancement of the production of regulatory T cells (Tregs) (11). CCHE1 has been shown to be functionally correlated with ERK/COX-2 pathway (12). Since COX-2 is expressed in response to inflammatory stimulators and participates in the inflammatory reactions (13), CCHE1 might be involved in the inflammatory responses. Based on the previous reports regarding the role of ANRIL, PICART1, MALAT1, CCAT1, CCAT2, and CCHE1 in the pathogenesis of immune-mediated disorders, we selected these lncRNAs to assess their potential as peripheral markers in the AIDP and CIDP.

## Materials and Methods

### Enrollment of Patients and Unaffected Controls

We recruited 22 patients with AIDP, 31 patients with CIDP, and 50 unaffected persons in the present investigation. Unaffected persons recruited as controls had no sign of immune-related conditions. The diagnosis of AIDP and CIDP was based on guidelines stated by the American Academy of Neurology (14) and the National Institute of Neurological Disorders and Stroke (15). Blood samples were taken from patients with AIDP and CIDP in their remission phase. Patients were not on any treatment at the time of sampling. Exclusion criteria were as follows: any recent or chronic infectious process, malignant conditions, and any systemic disorder. The study protocol was approved by the ethical committee of Shahid Beheshti University of Medical Sciences. All enlisted individuals signed the informed consent forms.

### Evaluation of the Expression of lncRNAs in Blood Samples

As the first step, RNA was recovered from blood samples using the commercial RNA extraction Kit (GeneAll, Seoul, South Korea). Then, a fraction of isolated RNA specimens was converted to complementary DNA (cDNA using the OneStep RT-PCR Series Kit (BioFact™, Seoul, South Korea). Expressions of mentioned lncRNAs were quantified in specimens using the RealQ Plus 2x Master Mix (Amplicon, Denmark). Transcript levels of the B2M gene were quantified and used for normalization of transcript levels of lncRNAs. All experiments were executed in duplicate. Primers were designed by Primer3 tool (https://primer3.ut.ee/). The specificity of primers was appraised by *in silico* tools. PCR products were also run on 2% agarose gel to verify the presence of expected amplicons and their sizes. Each run had a negative control comprising all PCR reagents except the template. Features of primers and amplified RNA parts are presented in Supplementary Material.

### Statistical Methods

R program was used for statistical analyses. Transcript quantities of lncRNAs in relation to the B2M gene were quantified using the following formula: $\frac{{\text{amp}}_{\text{gene}}^{-{\text{CT}}_{\text{gene}}}}{{\text{amp}}_{\text{B}2\text{M}}^{-{\text{CT}}_{\text{B}2\text{M}}}}$. Subsequently, we log2 transformed these values to compare their levels between study subgroups. The mean values of gene expression levels were compared between subgroups using the *t*-test. Correlations between RNA levels were judged using Spearman's correlation coefficients. Receiver operating characteristic (ROC) curves were depicted to appraise the diagnostic power of lncRNAs. At first, Bayesian generalized linear model, generalized linear model, and linear discriminant analysis with 10-fold cross validation were used to measure the related parameters. Then, the best model was used for further estimations.

## Results

### Global Information About Enlisted Individuals

Global data of enlisted persons in the AIDP, CIDP, and control groups are denoted in Table 1.

**Table 1:** Global data of enlisted persons.

**Variables**	**Cases with AIDP**	**Cases with CIDP**	**Controls**
Female/male [no. (%)]	6 (27%)/16 (73%)	11 (35%)/20 (65%)	25 (50%)/25 (50%)
Age (mean ± SD, Y)	49.72 ± 14.6	50.5 ± 15.8	44.48 ± 2.3

### Expression Analyses

**Figure 1:**
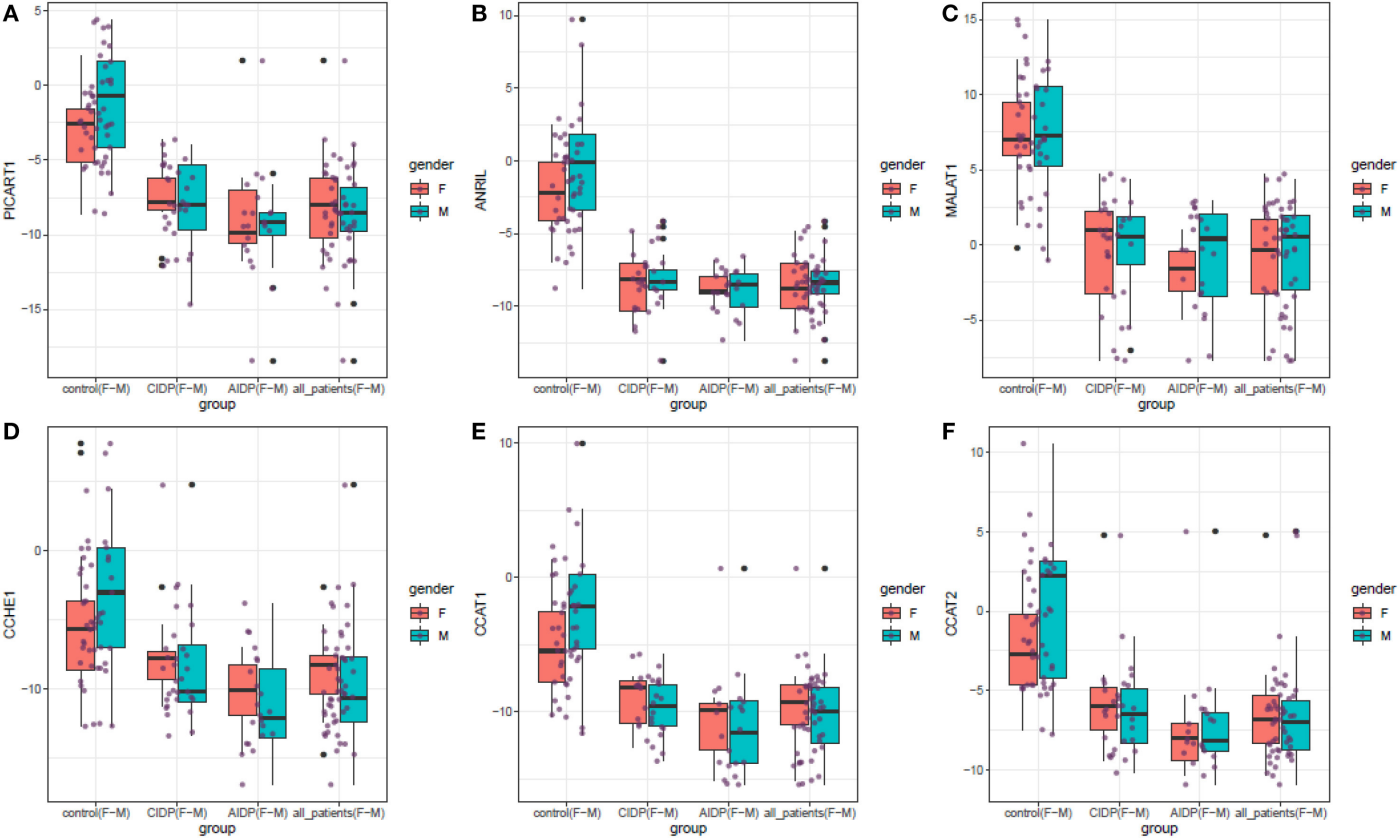
The relative quantification of the expression of ANRIL, PICART1, MALAT1, CCAT1, CCAT2, and CCHE1 lncRNAs in distinct study subgroups including the AIDP, CIDP, and controls. Upper/lower extremes, median, and upper/lower quartiles are shown. Each purple dot represents the gene expression level in one individual. Black dots show outliers (significance level: *p* < 0.05).

Figure 1 displays the relative quantification of ANRIL, PICART1, MALAT1, CCAT1, CCAT2, and CCHE1 lncRNAs levels in distinct study subgroups including the AIDP, CIDP, and controls.

ANRIL, PICART1, MALAT1, CCAT1, CCAT2, and CCHE1 lncRNAs were significantly downregulated in both cases with AIDP and CIDP compared with unaffected individuals. Gender-based comparisons also verified such downregulations in both male and female subjects compared with sex-matched unaffected controls for all lncRNAs. There was no significant difference in the expression of either lncRNA between cases with AIDP and cases with CIDP. Table 2 demonstrates the detailed parameters obtained from statistical analyses.

**Table 2:** Detailed statistics of the expression analysis of ANRIL, PICART1, MALAT1, CCAT1, CCAT2, and CCHE1 lncRNAs in AIDP, CIDP, and control groups.

		**PICART1**					**ANRIL**					**MALAT1**				
**Number of samples**		**SE**	**Ratio of mean expressions**	***P*-value**	**95% CI**		**SE**	**Ratio of mean expressions**	***P*-value**	**95% CI**		**SE**	**Ratio of mean expressions**	***P*-value**	**95% CI**	
**CIDP/Control**																
Total	31/50	0.67	0.02	**3.69E-13**	−7.24	−4.59	0.64	0.01	**2.89E-17**	−8.17	−5.63	0.83	0.00	**8.35E-14**	−9.51	−6.19
F	11/25	0.91	0.04	**5.79E-05**	−6.53	−2.74	0.86	0.01	**6.19E-07**	−8.06	−4.45	1.47	0.01	**1.06E-04**	−10.75	−4.48
M	20/25	0.96	0.01	**3.18E-09**	−9.02	−5.16	0.98	0.00	**3.06E-09**	−9.64	−5.66	1.09	0.00	**2.90E-09**	−10.35	−5.94
**AIDP/Control**																
Total	22/50	0.90	0.01	**1.31E-09**	−9.04	−5.41	0.60	0.01	**1.38E-19**	−8.67	−6.29	0.88	0.00	**1.06E-12**	−10.29	−6.77
F	6/25	2.10	0.04	6.77E-02	−9.95	0.48	0.68	0.01	**5.18E-08**	−7.81	−4.93	1.11	0.00	**5.23E-06**	−11.33	−6.45
M	16/25	1.03	0.00	**3.17E-10**	−10.89	−6.73	0.95	0.00	**3.02E-10**	−10.39	−6.51	1.22	0.00	**3.40E-08**	−11.10	−6.13
**CIDP/AIDP**																
Total	31/22	0.91	2.48	1.59E-01	−0.54	3.16	0.50	1.50	2.50E-01	−0.42	1.58	0.95	1.60	4.79E-01	−1.23	2.59
F	11/6	2.17	1.07	9.64E-01	−5.13	5.33	0.86	1.08	9.01E-01	−1.73	1.95	1.59	2.43	4.33E-01	−2.11	4.66
M	20/16	0.99	3.30	9.03E-02	−0.29	3.73	0.61	1.74	2.02E-01	−0.45	2.05	1.16	1.39	6.85E-01	−1.89	2.84
**All patients/controls**																
Total	53/50	0.63	0.01	**2.31E-17**	−7.71	−5.21	0.57	0.01	**5.71E-20**	−8.28	−6.01	0.71	0.00	**6.69E-20**	−9.54	−6.72
F	17/25	0.97	0.04	**4.83E-05**	−6.66	−2.68	0.68	0.01	**2.73E-11**	−7.68	−4.92	1.12	0.00	**3.57E-08**	−10.35	−5.78
M	36/25	0.87	0.00	**7.70E-12**	−9.60	−6.11	0.92	0.00	**9.30E-10**	−9.89	−6.13	1.00	0.00	**9.52E-11**	−10.36	−6.35
		**CCHE1**					**CCAT1**					**CCAT2**				
**Number of samples**		**SE**	**Ratio of mean expressions**	***P*****-value**	**95% CI**		**SE**	**Ratio of mean expressions**	***P*****-value**	**95% CI**		**SE**	**Ratio of mean expressions**	***P*****-value**	**95% CI**	
**CIDP/Control**																
Total	31/50	0.96	0.07	**1.48E-04**	−5.74	−1.92	0.73	0.02	**5.84E-11**	−7.00	−4.10	0.75	0.03	**1.01E-09**	−6.69	−3.72
F	11/25	1.26	0.22	9.27E-02	−4.76	0.38	0.86	0.06	**4.32E-05**	−5.85	−2.35	1.26	0.11	**2.45E-02**	−5.88	−0.47
M	20/25	1.29	0.03	**1.84E-04**	−7.91	−2.70	1.13	0.01	**5.62E-07**	−9.25	−4.64	1.01	0.01	**5.01E-08**	−8.99	−4.88
**AIDP/Control**																
Total	22/50	1.00	0.01	**2.79E-08**	−8.43	−4.44	1.00	0.01	**4.36E-09**	−9.20	−5.17	0.87	0.01	**2.02E-09**	−8.15	−4.65
F	6/25	1.56	0.04	**1.04E-02**	−7.97	−1.27	1.24	0.02	**9.62E-04**	−8.83	−3.19	0.92	0.02	**8.92E-05**	−7.80	−3.72
M	16/25	1.24	0.00	**2.51E-07**	−10.42	−5.38	1.44	0.00	**1.03E-06**	−11.36	−5.51	1.26	0.01	**7.44E-07**	−10.05	−4.94
**CIDP/AIDP**																
Total	31/22	0.99	6.05	**1.17E-02**	0.60	4.59	0.87	3.09	7.18E-02	−0.15	3.41	0.85	2.29	1.67E-01	−0.52	2.91
F	11/6	1.39	5.40	1.13E-01	−0.70	5.57	1.22	3.77	1.54E-01	−0.89	4.72	1.38	6.00	8.11E-02	−0.36	5.53
M	20/16	1.34	6.03	6.05E-02	−0.12	5.31	1.14	2.82	2.04E-01	−0.87	3.86	1.02	1.47	5.88E-01	−1.54	2.66
**All patients/controls**																
Total	53/50	0.86	0.03	**1.30E-07**	−6.62	−3.21	0.74	0.01	**8.95E-13**	−7.71	−4.75	0.68	0.02	**6.54E-13**	−7.06	−4.34
F	17/25	1.23	0.12	**1.77E-02**	−5.53	−0.56	0.85	0.04	**1.53E-06**	−6.48	−3.06	0.98	0.06	**2.66E-04**	−6.09	−2.08
M	36/25	1.10	0.01	**2.95E-07**	−8.66	−4.26	1.15	0.01	**9.04E-08**	−9.94	−5.27	1.01	0.01	**1.75E-08**	−9.22	−5.14

*Bold values mean item with significant difference among two groups*.

**Figure 2:**
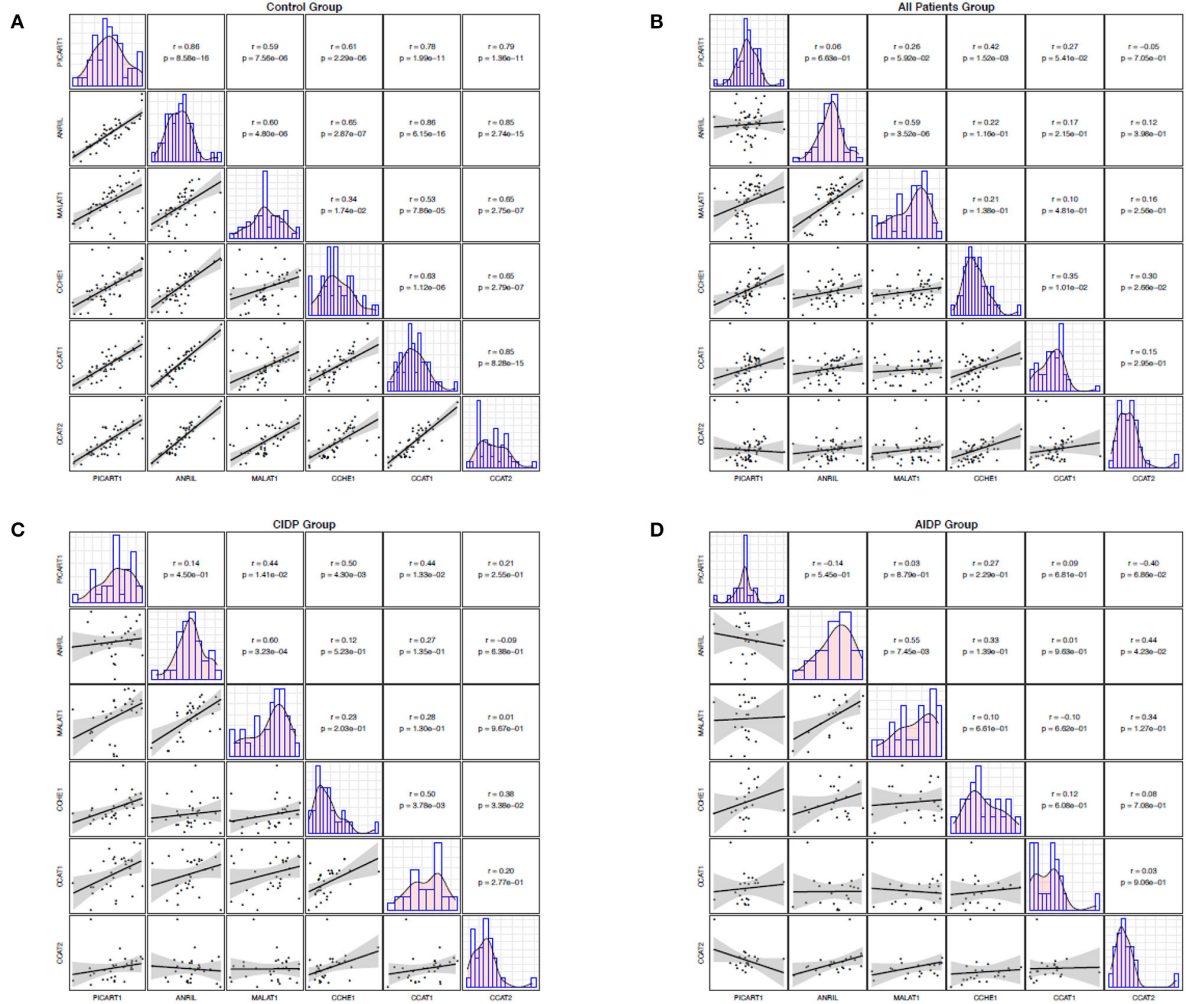
Correlation between the expression levels of ANRIL, PICART1, MALAT1, CCAT1, CCAT2, and CCHE1 lncRNAs in control group **(A)**, all patients **(B)**, CIDP **(C)**, and AIDP **(D)**. The lower panel shows scatter plots and smoothing splines for the expression levels of every possible pair of genes. The upper panel shows the corresponding Pearson's correlation coefficients and *P* values.

While the expression levels of ANRIL and PICART1 were significantly correlated in healthy subjects (r = 0.86, *p* = 8.5E-16), similar analysis in cases with AIDP and CIDP revealed no significant correlation. Among healthy subjects, correlation coefficients ranged from 0.86 between ANRIL and PICART1 to 0.34 between MALAT1 and CCHE1. In patients, the most robust correlation among patients was detected between ANRIL and MALAT1 lncRNAs (r = 0.59, *p* = 3.52E-6), while the minimum correlation coefficient was detected between ANRIL and PICART1 (r = 0.06, *p* = 6.63e-1). In cases with CIDP, correlation coefficients ranged from 0.01 (MALAT1/CCAT2) to 0.50 (PICART1/CCHE1 and CCAT2/CCHE1). Finally, among cases with AIDP, correlation coefficients ranged from 0.01 (ANRIL/CCAT2) to 0.55 (ANRIL/MALAT1). Figure 2 demonstrates correlation coefficients between lncRNAs pairs and significance levels in distinct study subgroups.

### Receiver Operating Characteristic Curves

**Figure 3:**
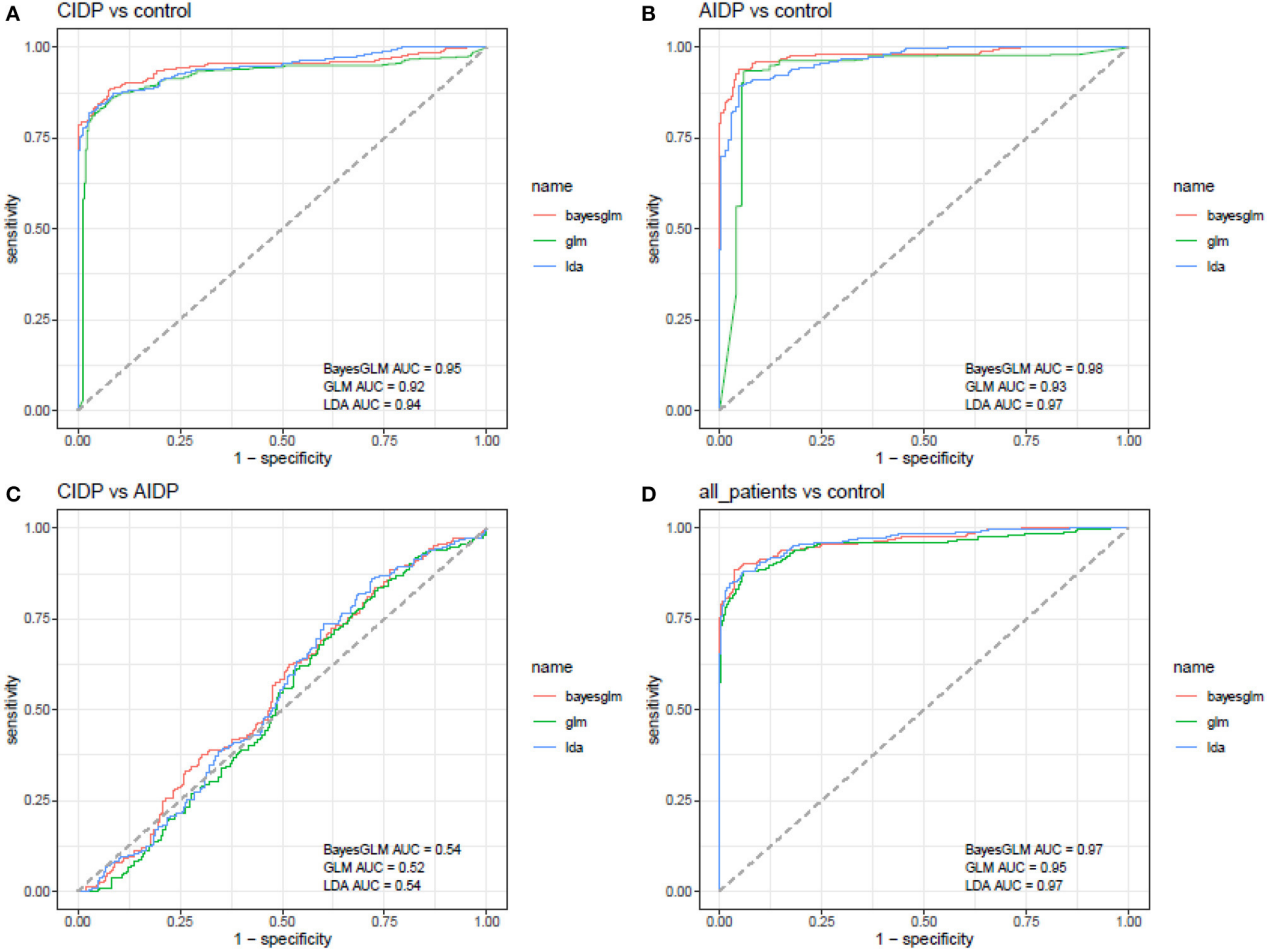
ROC curves depicted using Bayesian generalized linear model, generalized linear model, and linear discriminant analysis (AUC, area under curve).

**Figure 4:**
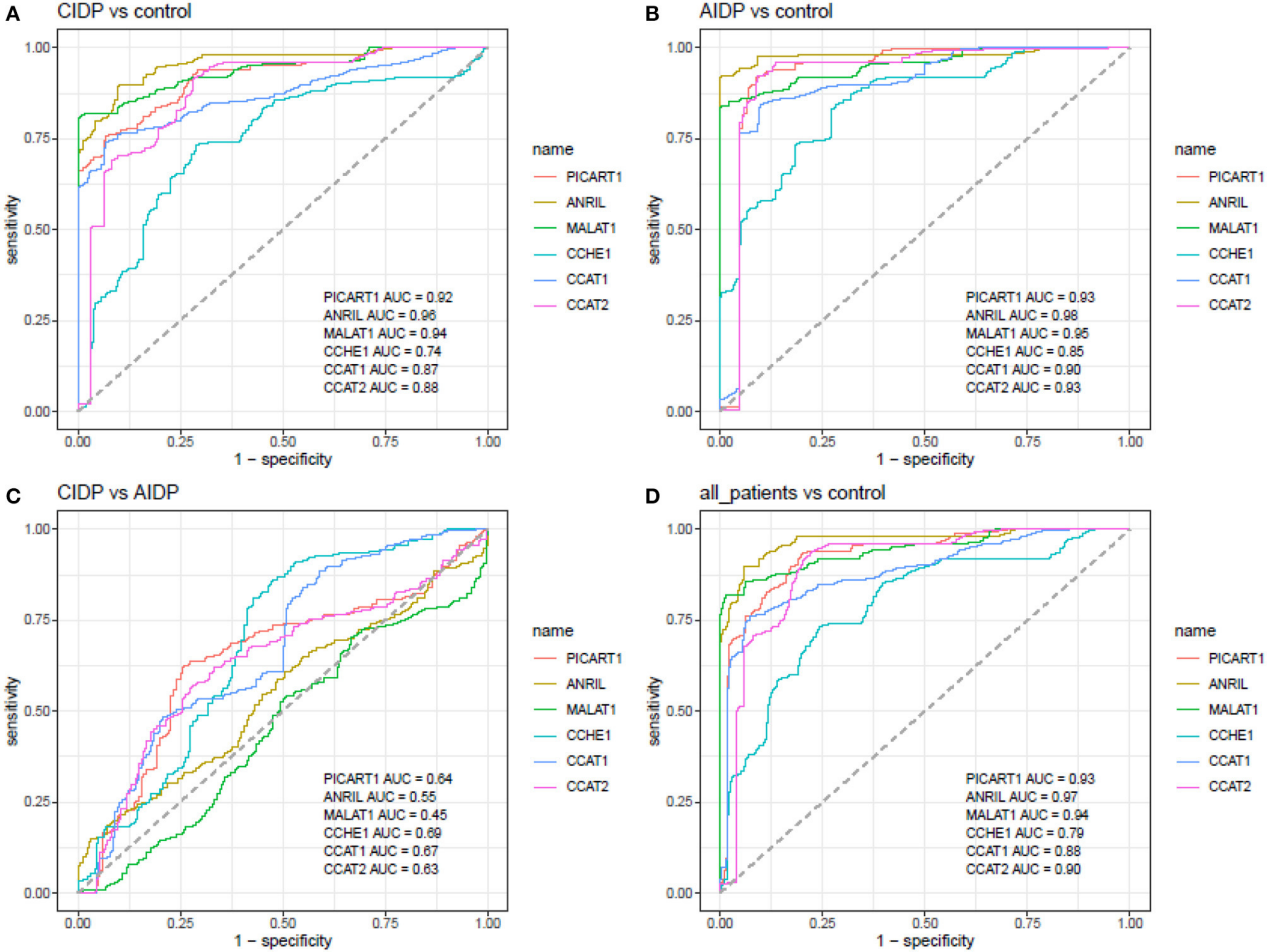
ROC curves depicted using the best model for estimation of diagnostic power of ANRIL, PICART1, MALAT1, CCAT1, CCAT2, and CCHE1 lncRNAs in the differentiation between the AIDP, CIDP, and control subjects (AUC, area under curve).

Receiver operating characteristic curves were depicted using Bayesian generalized linear model, generalized linear model, and linear discriminant analysis (Figure 3). Then, the best model was used for further estimations (Figure 4).

ANRIL, MALAT1, and PICART1 had the diagnostic power of 0.96, 0.94, and 0.92 in distinguishing between cases with CIDP and controls, respectively. A combination of all lncRNAs resulted in 0.95 diagnostic power with a sensitivity of 0.85 and specificity of 0.96 for this purpose. Diagnostic power values of these lncRNAs in the differentiation between cases with AIDP and controls were 0.98, 0.95, and 0.93, respectively. The combinatorial diagnostic power reached 0.98 for the differentiation between cases with AIDP and controls. The six-lncRNA panel could differentiate combined cases with AIDP and CIDP from controls with AUC, sensitivity, and specificity values of 0.97, 0.90, and 0.96, respectively (Table 3).

**Table 3:** Detailed statistics of ROC curves for the estimation of diagnostic power of ANRIL, PICART1, MALAT1, CCAT1, CCAT2, and CCHE1 lncRNAs in the differentiation between the AIDP, CIDP, and control subjects.

		**PICART1**			**ANRIL**			**MALAT1**			**CCHE1**			**CCAT1**			**CCAT2**			**All markers**		
**Number of samples**		**AUC**	**Sensitivity**	**Specificity**	**AUC**	**Sensitivity**	**Specificity**	**AUC**	**Sensitivity**	**Specificity**	**AUC**	**Sensitivity**	**Specificity**	**AUC**	**Sensitivity**	**Specificity**	**AUC**	**Sensitivity**	**Specificity**	**AUC**	**Sensitivity**	**Specificity**
**CIDP/Control**																						
Total	31/50	0.92	0.76	0.93	0.96	0.90	0.90	0.94	0.81	0.99	0.74	0.72	0.72	0.87	0.74	0.93	0.88	0.92	0.72	0.95	0.85	0.96
**AIDP/Control**																						
Total	22/50	0.93	0.91	0.95	0.98	0.92	1.00	0.95	0.83	1.00	0.85	0.74	0.81	0.90	0.85	0.90	0.93	0.96	0.88	0.98	0.94	0.97
**CIDP/AIDP**																						
Total	31/22	0.64	0.61	0.74	0.54	0.62	0.50	0.50	0.58	0.54	0.68	0.91	0.48	0.67	0.90	0.40	0.64	0.62	0.70	0.55	0.85	0.30
**All patients/controls**																						
Total	53/50	0.93	0.93	0.81	0.97	0.90	0.94	0.95	0.81	1.00	0.79	0.74	0.76	0.88	0.75	0.94	0.90	0.94	0.78	0.97	0.90	0.96

## Discussion

Based on the importance of finding biomarkers for immune-mediated neuropathies, we investigated the expression patterns of a panel of lncRNAs in patients with acute and chronic forms of this condition. Notably, we detected remarkable downregulation of ANRIL, PICART1, MALAT1, CCAT1, CCAT2, and CCHE1 in cases with both AIDP and CIDP compared with unaffected individuals. The significance of downregulation of some of these lncRNAs in the modulation of immune responses has been verified in other disorders. For instance, PICART1 suppression has been shown to result in JAK2/STAT3 activation (6). STAT3 has a critical role in the regulation of innate and adaptive immune reactions *via* modulating the expression of numerous cytokines such as interferons and interleukins−2,−6,−10, and−12 (16). The over-activation of STAT3 has a prominent role in the induction of autoimmune reactions in a wide range of tissues (17). Therefore, downregulation of PICART1 might participate in the pathogenesis of immune-mediated neuropathies through the activation of STAT3. Moreover, PICART1 interacts with AKT/GSK3β/β-catenin signaling pathway (18). Notably, GSK-3 and β-catenin regulate the innate immune response against RNA and DNA viruses (19). Based on the importance of viral infections in the initiation of AIDP (20), the interplay between PICART1 and these two proteins might be implicated in the pathogenesis of this form of immune-related neuropathies. MALAT1 has been shown to induce the expansion of tolerogenic DCs and Tregs through the miR155/DC-SIGN/IL-10 axis (21). Both mentioned cell populations contribute to the modulation of immune responses during the course of immune-related neuropathies (22, 23). Thus, this axis might also mediate the role of MALAT1 in the pathobiology of these conditions. CCAT1 has been shown to interact with miR-375-3p to decrease the expression of IRF5 (24), a transcription factor that controls the expression of several genes participating in the inflammatory reactions and induction of the immune responses (25). Therefore, the downregulation of *CCAT1* might influence the pathogenesis of AIDP or CIDP *via* this route. The expression of *CCHE1* has been correlated with the COX-2 pathway (12). The expression of COX-2 by macrophages might participate in the production of prostaglandins during the acute phase of demyelination in immune-related neuropathies (26). Therefore, the downregulation of CCHE1 in patients with immune-related neuropathies might be a compensative mechanism for amelioration of COX-2-related demyelinating effects in the acute phase. This speculation is supported by the fact that recruited patients in the current study were in the remission phase.

Although the functional consequences of the ANRIL downregulation have not been evaluated in the context of immune-related disorders, decreased levels of this lncRNA have also been noted in the peripheral blood of patients with periodontitis, a condition which is caused by the interplay between immune reactions and pathogens (27). The expression of ANRIL has been shown to be regulated by TNF-α (5), a cytokine with dual roles in the pathogenesis of immune-related neuropathies (28). Therefore, the functional relevance of the ANRIL downregulation should be appraised in these conditions.

Gender-based comparisons also verified the observed downregulations of lncRNAs in both male and female subjects compared with sex-matched unaffected controls. Therefore, the contribution of these lncRNAs in the pathogenesis of immune-mediated neuropathies is sex-independent. Moreover, there was no significant difference in the expression of either lncRNA between cases with AIDP and cases with CIDP, suggesting shared molecular mechanisms for these conditions in the terms of participation of mentioned lncRNAs.

The assessment of pairwise correlations between lncRNAs revealed distinct patterns among patients and healthy subjects. For instance, while the expression levels of ANRIL and PICART1 were significantly correlated in the healthy subjects, similar analysis in the cases with AIDP and CIDP revealed no significant correlation. The most robust correlation among patients was detected between ANRIL and MALAT1 lncRNAs. These findings indicate the influence of the disease condition on the correlation network between lncRNAs. Moreover, the altered interactions between lncRNAs might be involved in the pathogenesis of immune-related neuropathies.

The most important finding of our study has been the robust ability of the mentioned lncRNAs in distinguishing between patients with immune-related neuropathies and healthy status. The best values have been obtained for ANRIL, MALAT1, and PICART1. A combination of all lncRNAs resulted in 0.95 diagnostic power with a sensitivity of 0.85 and specificity of 0.96 for this purpose. These values are superior to presently used markers in this disorder. Most notably, the six-lncRNA panel could differentiate cases from controls with AUC, sensitivity, and specificity values of 0.97, 0.90, and 0.96, respectively. Therefore, we suggest this lncRNA panel as a sensitive and specific diagnostic panel for acquired immune-mediated polyneuropathies.

## Data Availability Statement

The raw data supporting the conclusions of this article will be made available by the authors, without undue reservation.

## Ethics Statement

The studies involving human participants were reviewed and approved by IR.SBMU.MSP.REC.1398.849. The patients/participants provided their written informed consent to participate in this study.

## Author Contributions

BH and FN performed the experiment. AS and HH analyzed the data. SG-F and MT wrote the draft and revised it. All authors approved the submitted version.

## Conflict of Interest

The authors declare that the research was conducted in the absence of any commercial or financial relationships that could be construed as a potential conflict of interest.
