# Supplementary material for: A Diagnostic Panel for Acquired Immune-Mediated Polyneuropathies Based on the Expression of lncRNAs
Source: Front Immunol. 2021 Feb 23;12:643615. doi: 10.3389/fimmu.2021.643615 (PMC7940672; doi:10.3389/fimmu.2021.643615)
Supplement: Supplementary file 1 [file Table_1.DOCX]

Table S1. Features of primers and amplified RNA parts.

| Gene | Primer | Sequence | Product length |
| --- | --- | --- | --- |
| *ANRIL* | Forward  Reverse | TGCTCTATCCGCCAATCAGG  GCGTGCAGCGGTTTAGTTT | 108 bp |
| *PICART1* | Forward  reverse | AGGCAGCTACTGTAATAAT GTACCCTGGGCCTTTCTTAC | 75 bp |
| *MALAT1* | Forward  Reverse | GACGGAGGTTGAGATGAAGC  ATTCGGGGCTCTGTAGTCCT | 84 bp |
| *CCAT1* | Forward  Reverse | GCCGTGTTAAGCATTGCGAA  TCATGTCTCGGCACCTTTCC | 168 bp |
| *CCAT2* | Forward  Reverse | AAGAGGGAGGTATCAACAGAGAC TTTGGACGACGCCTTCATTTC | 180 bp |
| *CCHE1* | Forward  reverse | AAGGTCCCAGGATACTCGC  GTGTCGTGGACTGGCAAAAT | 140 bp |
| *B2M* | Forward  Reverse | AGATGAGTATGCCTGCCGTG  GCGGCATCTTCAAACCTCCA | 105 bp |
